# Serum levels of brain-derived neurotrophic factor (BNDF) in multiple sclerosis patients with *Trichuris suis* ova therapy

**DOI:** 10.1051/parasite/2013056

**Published:** 2013-12-19

**Authors:** Berit Rosche, Jonas Werner, Friderike Joëlle Benzel, Lutz Harms, Heidi Danker-Hopfe, Rainer Hellweg

**Affiliations:** 1 Department of Neurology and Experimental Neurology, Charité Campus Mitte – Universitätsmedizin Berlin Germany; 2 Department of Psychiatry, Charité Campus Mitte – Universitätsmedizin Berlin Germany; 3 Competence Centre for Sleep Medicine, Charité Campus Benjamin Franklin – Universitätsmedizin Berlin Germany

**Keywords:** Multiple sclerosis, *Trichuris suis* ova, Brain-derived neurotrophic factor, Neuroprotection

## Abstract

We previously analysed clinical and immunological parameters under *Trichuris suis* ova (TSO) therapy in four patients with secondary progressive multiple sclerosis. The serum Brain-derived neurotrophic factor (BDNF) levels of these four patients were assessed before, during and after therapy with TSO and showed significant decrease of BDNF during TSO therapy (*p* < 0.05).

## Abstract (fr)

Nous avons précédemment analysé les paramètres cliniques et immunologiques sous thérapie par des œufs de *Trichuris suis* (TSO) chez quatre patients avec sclérose en plaques progressive secondaire. Les niveaux du facteur neurotrophique dérivé du cerveau (BNDF) chez ces quatre patients ont été mesurés avant, pendant et après la thérapie par TSO et ont montré une diminution significative du BNDF pendant la thérapie par TSO (*p* < 0.05).

## Introduction

Brain-derived neurotrophic factor (BDNF), a member of the neurotrophin protein family, is an important modulator of neurotransmitter release and synaptic plasticity [11, 16] and has been hypothesized to play a role in the neuroprotective mechanisms of some MS therapies [1, 8, 13].

Correale and Farez could demonstrate beneficial immunomodulation by helminths in humans in an observational study of relapsing-remitting multiple sclerosis (RRMS) patients with community-acquired gastrointestinal infections [3]. They demonstrated that B cells isolated from these helminth-infected MS patients produced greater amounts of BDNF *in vitro* compared to those of normal subjects [4]. Fleming and colleagues reported favourable MRI and immunological results in MS patients after probiotic treatment with *Trichuris suis* ova (TSO) [5].

In the small trial presented previously we analysed the effects of TSO therapy on disease course and different immunological parameters in four patients with secondary progressive MS and found downregulation of Th1-associated cytokines [2]. With the present paper we analysed in this four patients the role of BDNF in MS therapy using TSO.

## Material and methods

Four patients with secondary progressive MS without any other immunomodulatory or immunosuppressive therapy were treated for 6 months with TSO 2,500 eggs orally every 14 day. TSO were produced and provided by Ovamed GmbH, Barsbüttel, Germany, as a non-sterile aqueous suspension containing 2,500 embryonated alive TSO in 15 mL of sulphate stabilizer (0.05 mmol/l H_2_SO_4_). Patients were recruited from the Department of Neurology, Charité – Universitätsmedizin Berlin, and all fulfilled the 2005 McDonald criteria [12]. The study was approved by the local ethics committee. Blood for BDNF analysis was drawn in the morning, centrifuged (800 g for 15 min) and stored at −80 °C until the BDNF concentrations were determined. BDNF serum concentration was quantified by a modified ELISA (Promega Co., Madison, WI, USA) as previously described [15].

To evaluate effects of TSO therapy on BDNF serum levels we used a *t*-test for paired observations.

Serum from patients was available 1 month before (−1), at the start (0), and at months 1, 2, 3, 6 during therapy, as well as 1 and 4 months after TSO therapy.

## Results

BDNF serum levels were within the range of previously published data on healthy controls [7]. When calculating means of BDNF levels from our four patients for each time point we saw a clear decrease under treatment and in the post-treatment period compared to the pre-treatment period (month −1: 3682.55 ± 1105.17; month 0: 3938.15 ± 572.08; month 1: 3744.98 ± 710.07; month 2: 2876.92 ± 381.10; month 3: 3238.18 ± 885.93; month 6: 2765.28 ± 1236.73; month 7: 2922.97 ± 615.90; month 10: 2826.03 ± 569.61). Statistical analysis of BDNF levels of different time points by *t*-test failed significance due to small sample size of this preliminary study.

**Figure 1.**
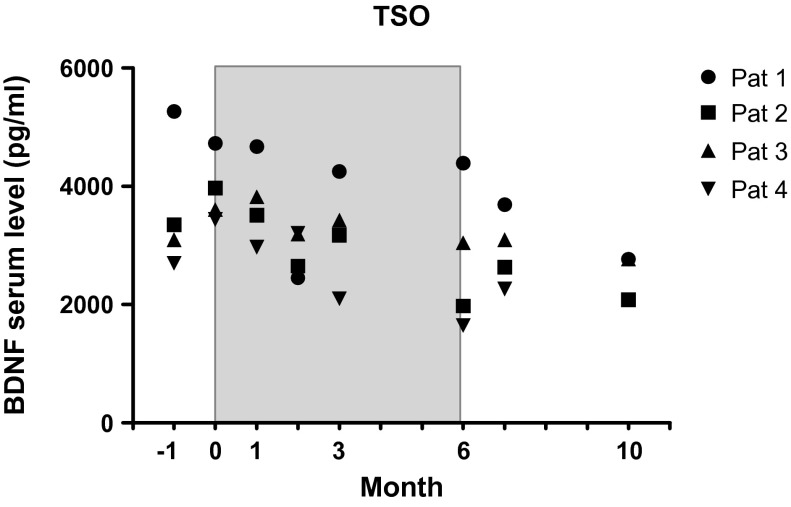
BDNF serum levels of SPMS patients prior to (Month –1, 0), during (Months 1, 2, 3, 6) and subsequent to (Month 7, 10) therapy with TSO (shaded area).

Because the BDNF levels did not differ significantly between the two pre-treatment measurements, a mean pre-treatment BDNF level was computed at the individual level. The same procedure was applied for the two post-treatment levels, which also did not differ significantly from each other. A pairwise comparison of the four BDNF levels under treatment revealed statistically significant differences only between the first and the last assessment (*p* < 0.05). The BDNF levels after therapy were significantly lower than before therapy: pre-treatment: 3810.4 ± 831.1; post-treatment: 2875.5 ± 572.6, *p* < 0.02 (see Figure 1).

## Discussion

Taken together, we found a significant decrease in serum levels of BDNF in our four SPMS patients under therapy with TSO and speculate that TSO influences BDNF metabolism.

Assessment of serum BDNF levels in MS has yielded conflicting results to date [1, 6, 10]. Besides by neuronal secretion BDNF can also be produced by activated T cells, B cells, monocytes and macrophages and is influenced by handling of blood ex vivo [9]. Especially the BDNF production of immune cells in inflammatory brain lesions is discussed to be a result of body’s compensatory neuroprotective mechanism. In line with this understanding is the increase of BDNF levels during relapses in MS [6] and in stimulated immune cells of patients with higher inflammatory activity in the white matter [14].

Correale and colleagues showed increased production of BDNF and nerve growth factor in stimulated B cells from MS patients with a helminth infection compared to uninfected patients and controls. The authors discuss that these neurotrophic factors, produced by B cells, might have a possible immunoregulatory and probably immunosuppressive effect on T cells in helminth-infected MS patients.

An explanation for the opposite trends in BDNF levels shown in the study of Correale and ours may be several differences in study design: e.g. relapsing-remitting vs. secondary progressive MS, stimulated B cells vs. serum levels, natural infections vs. experimental TSO treatment, clinical observational vs. prospective clinical trial study design. In our case, the decreased serum BDNF levels under helminth therapy led us to speculate that reduced inflammatory activation of the CNS caused the decrease in serum BDNF levels. Because peripheral immune cells can also be a source of peripheral BDNF, one could discuss, that TSO induces reduced inflammatory activation of peripheral immune cells and consequently a reduced serum BDNF level.

One limitation of this pilot study is the small sample size of the trial, which was one of the first investigating TSO treatments in MS.

Although the precise relationship between serum BDNF levels and cerebral concentration of BDNF in humans remains unclear, our study does not suggest that BDNF might have a neuroprotective effect in MS patients treated with TSO.
